# Special Issue: Processing, Structure, Dynamics and Mechanical Properties of Polymeric Materials

**DOI:** 10.3390/ma15093143

**Published:** 2022-04-26

**Authors:** Janusz W. Sikora

**Affiliations:** Department of Technology and Polymer Processing, Faculty of Mechanical Engineering, Lublin University of Technology, 36, Nadbystrzycka St., 20-618 Lublin, Poland; janusz.sikora@pollub.pl; Tel.: +48-81-5384221

The current Special Issue entitled “Processing, structure, dynamics and mechanical properties of polymeric materials” brings together scientists working at universities, research institutes, laboratories and various industries to discuss cutting-edge research on processing new polymeric materials using standard and innovative machines and to understand the structure and properties of these materials. New challenges related to the need to develop new biodegradable materials with new properties and structures, which are difficult to process by conventional processing methods, also concern the progress and development of machines, especially the most common ones, such as extruders and injection moulding machines. The aim of the Special Issue is to collect current research and analyses concerning the properties and structure of new polymeric materials of various applications, with special emphasis on biodegradable materials, and to show the possibilities of applying optimisation methods in polymer processing, which can be confidently applied with very good results and without large computational effort. In the opinion of the authors, the presented collection of papers is a fragment of contemporary, interesting directions and development trends in polymer processing.

The Guest Editors would like to emphasise their immense gratitude to the Editors-in-Chief of *Materials* magazine for the fantastic opportunity to manage this Special Issue. Likewise, to all of the authors from many different countries (Poland, the UK, China, Slovakia, the Czech Republic, Ukraine, Korea, Portugal) who have contributed to the success of this Special Issue, we thank them for their very high-quality work. Mention should also be made of the reviewers who provided support with insightful comments that undoubtedly improved the quality of the submitted papers. Finally, thanks are due to the Managing Editor of the Section, Ms Yulia Zhao, for her thorough and constant guidance of the editorial process. The success of this endeavour is evidenced by the 14 papers collected and published in two years. A brief overview of these papers is provided below to highlight the multidisciplinary nature and quality of this research.

Sikora et al. [1,2] characterised the plastic extrusion process and selected properties of three types of biodegradable plastics in comparison to LDPE. They described the properties of films obtained from thermoplastic potato starch (TPS-P), thermoplastic corn starch (TPS-C) and polylactic acid plastic (PLA) in comparison with those of LDPE. The produced plastic films were tested to determine the geometric characteristics, MFR, blow-up ratio, pull-down ratio, mass flow rate and exit velocity. The study was supplemented by thermogravimetry, differential scanning calorimetry and chemical structure analysis. It was found that the biodegradable films were extruded at a higher speed and mass flow rate than LDPE, film samples extruded from TPS-C and TPS-P showed the lowest thermal stability and all biodegradable plastics tested contained polyethylene. In addition, the breaking stress; breaking strain; static and dynamic friction coefficient of the film in longitudinal and transverse directions; puncture resistance and breaking strain; colour; brightness and gloss of the film; surface roughness; barrier properties; and microstructure were determined for each film. The studied biodegradable plastics show comparable or even better mechanical strength than petrochemical polyethylene in the range of applied processing parameters of film blowing. The effect of the screw speed on the mechanical properties of the films obtained was also demonstrated. As the screw speed increased, a decrease in the barrier’s properties was observed. No correlation was found between the roughness and gas and water vapour permeability. It was indicated that biodegradable plastics can compete with conventional petrochemical plastics used in blow moulding niches where the cost, recyclability, optical properties and water vapour barrier are not crucial.

Siegkas [3] described and implemented a computational method of generating porous materials and composites for additive manufacturing techniques using 3D Voronoi cells as space holders. The method makes it possible to potentially use the available materials to create hybrid metamaterials with tailored properties. The author pointed out that the macroscopic mechanical properties of the resulting structures can be tuned by controlling the microstructural features. The method is used to generate porous and composite structures with the use of polymer filaments, i.e., polylactic acid (PLA), thermoplastic polyurethane (TPU) and nylon. The method provides a series of variables that can potentially be manipulated to define the macroscopic properties of the material. The number, size and distribution of Voronoi cells; the percentage of filling during 3D printing; and the combination of materials can provide a number of resulting properties.

The applicability of physical methods to detect thermally degraded recycled material in polypropylene plastic parts was investigated by Běhálek et al. [4]. Standard methods of evaluating the mechanical properties of the material under static tensile and bending stress, as well as under dynamic impact stress using the Charpy method, were used for the experimental measurements. The rheological properties of the materials were monitored using a method involving measuring the melt flow index, while their thermal properties and oxidative stability were monitored using differential scanning calorimetry. They found that the most suitable technique for detecting thermally degraded recycled material in polypropylene is the method involving establishing the melt flow index. The bending test seems to be the most suitable method for detecting recycled material by measuring the material’s mechanical properties.

In turn, Grytsenko et al. [5] studied the copolymer structure and properties of 2-hydroxyethylmethacrylate (HEMA) with polyvinylpyrrolidone (PVP) and their hydrogels, obtained by block polymerisation in the presence of iron sulfate (II). They showed the correlation between the sorption-diffusion, physical-mechanical and thermophysical properties with the structure of the obtained materials. With the increase in the content of PVP in the original composition, the efficiency of its grafting and the cross-linking density of the polymer network decreased, while the surface hardness, heat resistance, sorption capacity of the copolymers in the dry state, ion permeability and elasticity in the swollen state increased and their tensile strength deteriorated. They proved that by changing the original composition formulation, it is possible to change the structure and thus the properties of the copolymers in the desired direction. A wavy physical structure with a periodicity that can impart flexibility to optoelectronic devices and control the optical path through a material stack has been affected by a low-pressure plasma of oxygen, argon, and nitrogen, as well as the power, flow rate and treatment time, as it was observed by Gu et al. [6].

They determined that increasing the power of the nitrogen and oxygen plasma increased the wavelengths and heights of the wrinkles; however, increasing the power of the argon plasma increased the wavelengths and decreased the heights of the wrinkles. On the basis of the results, it was concluded that a combination of different plasma gases could achieve exclusive control over either the wavelength or the height and allow a thorough analysis of the correlation between the wrinkle features and the characteristics of the electronic devices.

The aim of the work of Galej et al. [7] was to reduce warping of 3D prints made of polyoxymethylene (POM). The authors achieved this by modifying POM with ethylene vinyl acetate (EVA). The modifications performed decreased the stiffness and strength of the material, simultaneously enhancing its ductility. The lowering of the melting point and the slight increase in thermal stability with the addition of EVA widened the processing window for 3D printing. Trials of 3D printing on two different printers showed that the addition of EVA copolymer increased the incidence of successful printing without defects, making room for further development.

In turn, Moravskyi et al. [8] proposed a method of introducing a metal filler into the polymer matrix with the help of a chemical metallisation of the surface of the polymer raw material. The method was found to be effective and can be used to obtain high-tech metal-filled polymer composites, including those based on waste polymeric materials. They found that the creation of a metal shell on the polymer surface, which is destroyed during the melting of the polymer, ensures easy introduction and uniform distribution of the metal over the volume of the material. Their research proved that the introduction of metal into the polymer matrix in the form of a metal coating formed on the polymer surface guarantees the production of metal-containing polymer composites, which are characterised by uniform distribution of metal in the polymer matrix and high technological and operational properties.

In [9], He et al. prepared blends consisting of compatibilisers of different weight percentages and poly(lactic acid) (PLA) at 75 wt% and poly(butylene adipate-co-terephthalate) (PBAT) at 25 wt%. For this purpose, they used a self-made parallel three-screw extruder to induce an intense shearing flow. The impact resistance, compatibility and microscopic morphology of different blends with the same compatibiliser content gradient were studied and compared. They showed that the compatibilisers reacted with the hydroxyl groups of PLA or PBAT, causing, in the case of one compatibiliser, a significant increase in impact resistance, which indicates a further direction of research to improve the properties of PLA.

Bukala et al. [10] proposed a new approach to model constitutive bioresorbable polymers such as poly(lactic-co-glycolic acid) (PLGA), which is based on an isotropic elastic-low-plastic model (Cowper-Symonds). The use of PLGA in coronary stents can reduce the risk of complications (e.g., restenosis, thrombosis) after percutaneous coronary intervention. This approach was validated by two separate experimental studies. The correlation found between the numerical and experimental curves was satisfactory, proving the accuracy of the approach taken and making the model a useful tool for both the design and evaluation of, e.g., bioresorbable stents.

Zhang et al. [11] carried out studies on simultaneous thermal stretched polyamides by changing the structure and morphology of γ-PA1010 films prepared by melt-quenching. They found that the crystallinity has a significant effect on the film properties and that it can be influenced by changing the temperature and tensile force.

The static and dynamic mechanical properties of 3D-printed acrylonitrile butadiene styrene (ABS) as a function of the raster angle were considered by Galej et al. [12]. They proved that there exists an optimum raster angle that provides 3D objects with exceptional mechanical properties, especially impact strength and durability. They also confirmed that FDM still cannot be considered as a full-fledged alternative to injection moulding when it comes to the mechanical performance of the resulting products.

Finally, the review presented by Cunha et al. [13,14] evaluates the application of optimisation methodologies to the main polymer processing methods: single and twin-screw extruders, extrusion dies and calibrators, injection moulding, blow moulding and thermoforming technologies. The authors highlighted the most important characteristics related to the usage of optimisation techniques, such as the nature of the objective function, the type of optimisation algorithm, the modelling approach used to evaluate the solutions and the parameters to optimise. The aim of this extensive work was to identify the most important features of an optimisation system for polymer processing problems and to define the best procedure for each particular practical situation. It is concluded that there is a set of methodologies that can be confidently applied in polymer processing with very good performance and without the need of demanding computation requirements.
